# Bruton's Tyrosine Kinase (BTK) and Vav1 Contribute to Dectin1-Dependent Phagocytosis of *Candida albicans* in Macrophages

**DOI:** 10.1371/journal.ppat.1003446

**Published:** 2013-06-27

**Authors:** Karin Strijbis, Fikadu G. Tafesse, Gregory D. Fairn, Martin D. Witte, Stephanie K. Dougan, Nicki Watson, Eric Spooner, Alexandre Esteban, Valmik K. Vyas, Gerald R. Fink, Sergio Grinstein, Hidde L. Ploegh

**Affiliations:** 1 Whitehead Institute for Biomedical Research, Cambridge, Massachusetts, United States of America; 2 Keenan Research Centre of the Li Ka Shing Knowledge Institute, St. Michael's Hospital, Department of Surgery, University of Toronto, Toronto, Ontario, Canada; 3 Program in Cell Biology, Hospital for Sick Children, Toronto, Ontario, Canada; University of Birmingham, United Kingdom

## Abstract

Phagocytosis of the opportunistic fungal pathogen *Candida albicans* by cells of the innate immune system is vital to prevent infection. Dectin-1 is the major phagocytic receptor involved in anti-fungal immunity. We identify two new interacting proteins of Dectin-1 in macrophages, Bruton's Tyrosine Kinase (BTK) and Vav1. BTK and Vav1 are recruited to phagocytic cups containing *C. albicans* yeasts or hyphae but are absent from mature phagosomes. BTK and Vav1 localize to cuff regions surrounding the hyphae, while Dectin-1 lines the full length of the phagosome. BTK and Vav1 colocalize with the lipid PI(3,4,5)P_3_ and F-actin at the phagocytic cup, but not with diacylglycerol (DAG) which marks more mature phagosomal membranes. Using a selective BTK inhibitor, we show that BTK contributes to DAG synthesis at the phagocytic cup and the subsequent recruitment of PKCε. BTK- or Vav1-deficient peritoneal macrophages display a defect in both zymosan and *C. albicans* phagocytosis. Bone marrow-derived macrophages that lack BTK or Vav1 show reduced uptake of *C. albicans*, comparable to Dectin1-deficient cells. BTK- or Vav1-deficient mice are more susceptible to systemic *C. albicans* infection than wild type mice. This work identifies an important role for BTK and Vav1 in immune responses against *C. albicans*.

## Author Summary

The opportunistic yeast *Candida albicans* is a commensal organism of the human digestive tract, but also the most common cause of human fungal infections. Phagocytosis, the process by which innate immune cells engulf pathogens, is vital to prevent *C. albicans* infections. The major phagocytic receptor involved in anti-fungal immunity is Dectin-1. We identify two new interacting proteins of Dectin-1 in macrophages: Bruton's Tyrosine Kinase (BTK) and Vav1. In the course of phagocytosis, different phosphoinositides (PIs) are formed in the phagosomal membrane to allow the recruitment of proteins equipped with specialized lipid-interaction domains. We show that BTK and Vav1 colocalize with the lipid PI(3,4,5)P_3_ at the phagocytic cup, but not with diacylglycerol (DAG), which marks more mature phagosomal membranes. Inhibition of BTK affects the production of DAG and the recruitment of DAG-interacting proteins. BTK and Vav1 are essential for *C. albicans* immune responses, as BTK- or Vav1-deficient macrophages show reduced uptake of *C. albicans* and BTK- or Vav1-deficient deficient mice are more susceptible to systemic *C. albicans* infection. This work identifies an important role for BTK and Vav1 in immune responses against *C. albicans*.

## Introduction

Innate immune cells eliminate pathogens by phagocytosis, a process of internalization followed by degradation of the pathogen. Germline-encoded pattern recognition receptors (PRRs) recognize pathogen-associated molecular patterns (PAMPs) on bacteria, viruses, yeast and other microorganisms. Recognition of a PAMP by its receptor initiates a coordinated sequence of events that includes the recruitment of ancillary proteins and the formation of various second messengers at -or close to- the site of initial contact with the pathogen.

Macrophages and neutrophils are the first line of defense against *Candida albicans*, a common cause of human fungal infections [1]. *C. albicans* is an opportunistic commensal yeast that is part of the normal gut microbiota [2]. Innate immune cells must therefore tolerate commensal *C. albicans*, yet adequately deal with its pathogenic counterpart. The major PRR involved in anti-fungal immunity is Dectin-1, a C-type lectin present on neutrophils, macrophages and dendritic cells that recognizes fungal β-glucan. Dectin-1 contains an extracellular C-type lectin domain and an intracellular ITAM-like domain essential for downstream signaling [3]. Upon activation of Dectin-1, phosphorylation of its ITAM-like domain leads to the recruitment of spleen tyrosine kinase (Syk) [4]. The role of Syk in Dectin-1-mediated phagocytosis is cell type-dependent: Syk is essential for phagocytosis in dendritic cells, but not in macrophages [3], [4]. Other proteins that interact with Dectin-1 are PKCδ [5], the tetraspanin CD37 [6], Galectin-3 [7] and TLR2 [8]. Ectopic expression of Dectin-1 in fibroblasts or kidney cells confers phagocytic capacity to these cells [3], [7]. Dectin-1 is thus a bona fide phagocytic receptor, but the detailed mechanisms that underlie Dectin1-mediated phagocytosis are not known.

Actin drives phagocytosis: formation of the phagocytic cup depends on the formation of F-actin, and closure of the phagosome requires the reversal of actin polymerization [9]. In the course of FcγR-mediated phagocytosis -the best-understood model of phagocytosis-, several phosphoinositides (PI) are formed in the phagosomal membrane, which serve as docking stations for proteins with PI-specific interaction domains. Phosphatidylinositol 4,5-*bis*phosphate (PI(4,5)P_2_) is ubiquitously present in the plasma membrane and is transiently enriched in phagocytic cups [10]. As the cup forms, phosphatidylinositol 3-kinase (PI3K) converts PI(4,5)P_2_ to phosphatidylinositol 3,4,5-*tris*phosphate (PI(3,4,5)P_3_) which in turn can be converted to PI(3,4)P_2_ by the SH2-containing inositol 5′-phosphatase (SHIP). Proteins with a Pleckstrin Homology (PH) domain can bind to PI(4,5)P_2_, PI(3,4,5)P_3_ or PI(3,4)P_2_, allowing their recruitment to the maturing phagosome. In addition, phospholipase C (PLCγ) acts on PI(4,5)P_2_ to yield the second messengers IP_3_ and diacylglycerol (DAG). IP_3_ triggers an increase in cytoplasmic Ca^2+^ concentration, while DAG serves as a docking site for proteins with a C1 domain. The quantity, timing and localization of PI(3,4,5)P_3_, PI(3,4)P_2_ and DAG formation vary, depending on the phagocytic receptor and the identity of the particle engaged. Formation of PI(3,4,5)P_3_/PI(3,4)P_2_ and localization of actin to the phagocytic cup are also known to occur during phagocytosis of *C. albicans* by macrophages [11], [12] but downstream pathways engaged by the PI during Dectin-1 phagocytosis remain to be studied in detail.

Here we describe two new interactors of Dectin-1: Bruton's Tyrosine Kinase (BTK) and the guanine nucleotide exchange factor Vav1. We provide evidence that these proteins bind to PI(3,4,5)P_3_-rich membranes and that BTK is involved in the production of DAG during *C. albicans* phagocytosis. BTK and Vav1-deficient macrophages show reduced rates of phagocytosis and BTK and Vav1-deficient mice succumb more readily to *C. albicans* systemic infections than wild type mice.

## Results

### β-glucan exposure on *Candida albicans* yeasts and hyphae

**Figure 1:**
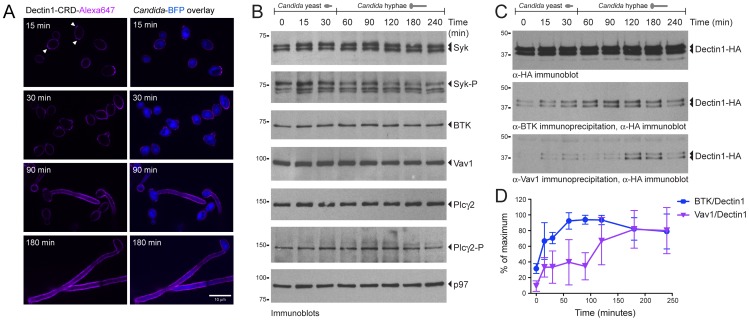
BTK and Vav1 interact with Dectin-1 during *C. albicans* phagocytosis by macrophages. (A): Morphology and β-glucan exposure of *C. albicans* expressing blue fluorescent protein (*Candida*-BFP) at indicated time points after incubation in DMEM with 10% IFS. *Candida*-BFP was stained with fluorescent carbohydrate recognition domain of Dectin-1 (Dectin1-CRD-Alexa647) that binds β-glucan. Arrows indicate increases staining at bud scars. (B): Immunoblotting experiment showing expression of different proteins in RAW-Dectin1 cells during co-incubation with live *C. albicans* for the indicated time points. Phagocytosis of *C. albicans* occurs throughout the time course, but the morphology of the ingested particles changes over time. (C): Co-incubation of RAW-Dectin1 with *C. albicans* followed by co-immunoprecipitation with anti-BTK or anti-Vav1 antibody and immunoblotting with anti-HA to detect Dectin-1. BTK/Dectin-1 and Vav1/Dectin-1 complexes were detected at different time points during the co-incubation. (D): Quantification of BTK/Dectin-1 and Vav1/Dectin-1 complexes showing strongest interactions at the 90- and 180-minute time points, respectively. Means +/− SD of three independent experiments are shown.

To facilitate imaging of phagocytosis, we applied two new imaging tools. First, we used a *Candida* strain that expresses a variant of blue fluorescent protein (*Candida*-BFP). Second, to study β-glucan exposure on *C. albicans* yeasts and hyphae, we site-specifically fluorescently labeled the extracellular carbohydrate recognition domain of Dectin-1 (Dectin1-CRD-Alexa647) using the bacterial enzyme sortase [7]. *Candida*-BFP was incubated in DMEM with 10% serum for 15, 30, 90 and 180 min followed by staining with Dectin1-CRD-Alexa647 (**Figure 1A**). At 15 and 30 min we observed moderately stained *C. albicans* yeasts with increased staining of bud scars (white arrows), congruent with increased β-glucan exposure at these sites [13]. At 90 and 180 min, formation of hyphae was extensive with strong, homogeneous β-glucan exposure. Under these growth conditions, when compared to *C. albicans* yeast, *C. albicans* hyphae thus expose higher levels of β-glucan, which is expected to affect signaling through Dectin-1.

### Identification of Dectin1-interacting proteins

To identify proteins that interact with Dectin-1 during phagocytosis of live *C. albicans*, we performed immuno-isolation experiments using the RAW-Dectin1 macrophage cell line (Esteban et al., 2011). The macrophages were co-incubated with live *C. albicans* for one hour, a time point that marks early hyphal formation and increased β-glucan exposure. Cells were lysed and Dectin-1, together with its interacting partners, was immunoprecipitated using anti-HA antibodies. Proteins present in these samples were analyzed and identified by SDS-PAGE, followed by LC-MS-MS. From the list of proteins, we selected two that were retrieved in complex with Dectin-1 in the samples with *C. albicans*, but that were absent from the control sample. These proteins were Bruton's Tyrosine Kinase (BTK) and the guanine nucleotide exchange factor Vav1.

To investigate the expression of BTK, Vav1 and other proteins already known to be involved in Dectin1-mediated phagocytosis, we incubated RAW-Dectin1 macrophages with live *C. albicans*. Phagocytosis continues throughout the period of coincubation, but *C. albicans* morphology changes from yeast form to hyphal form over time. Total cell lysates from the different time points were analyzed by immunoblot. Spleen tyrosine kinase (Syk), a known interactor of Dectin-1, was present at constant levels at all-time points (**Figure 1B**). Phosphorylation of Syk increased at 15 min and then waned. BTK and Vav1 were likewise present at constant levels. PLCγ2, a key enzyme in phagocytosis, was present at constant levels throughout the time course; its phosphorylation (at Y1217) was most pronounced around 90 and 120 min.

Next we confirmed the interaction between Dectin-1 and BTK, and between Dectin-1 and Vav1. Immunoprecipitation with polyclonal anti-BTK antibody, followed by immunoblotting with anti-HA, showed an increased interaction between BTK and Dectin-1 starting at 15 min, which gradually increased (**Figure 1C**). The interaction between Vav1 and Dectin-1 is strongest at the later time points (**Figure 1C**). Quantification of the Dectin-1/BTK and Dectin-1/Vav1 interactions using ImageJ software showed that the level of the Dectin-1/BTK complex peaks at 60–90 min, while the strongest Dectin-1/Vav1 interaction was observed at 180 min (**Figure 1D**). We conclude that BTK and Vav1 are expressed at constant levels, and that their interactions with Dectin-1 are strongest at the later time points when *C. albicans* has formed hyphae.

### Confirmation of BTK and Vav1 localization to the phagocytic cup

**Figure 2:**
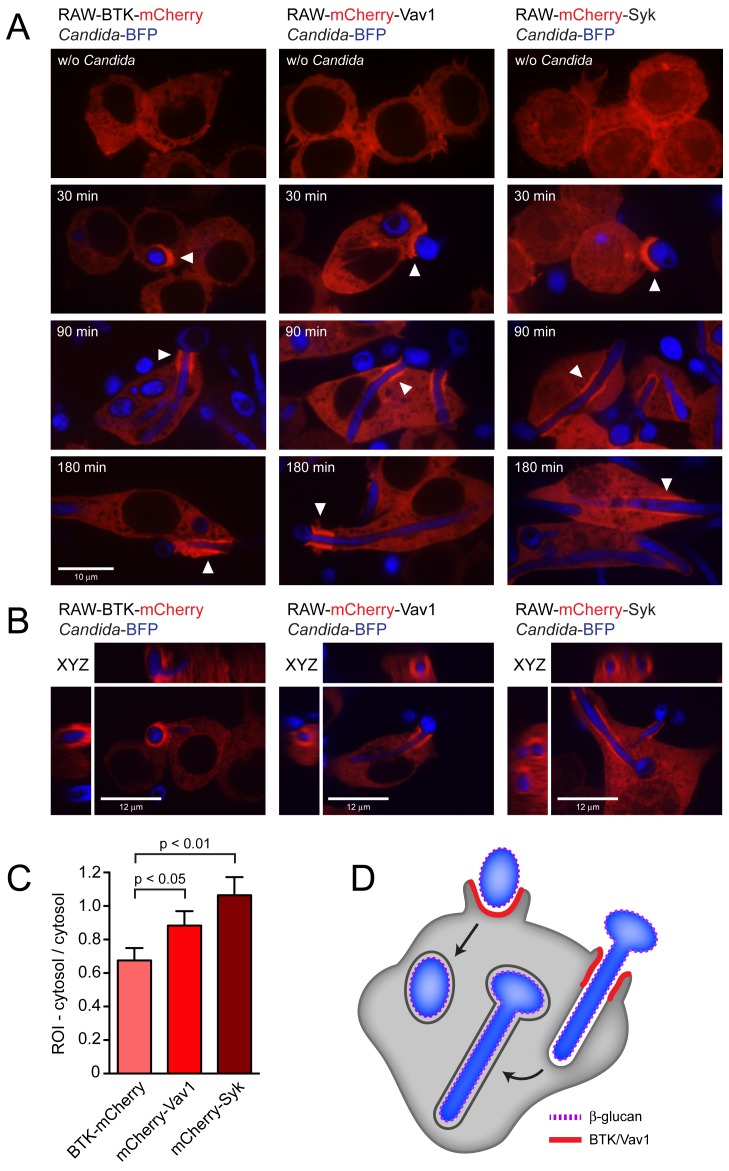
Localization of BTK-mCherry and mCherry-Vav1 to the *Candida*-containing phagocytic cup. (A): Confocal images showing localization of BTK-mCherry, mCherry-Vav1 and mCherry-Syk in RAW-Dectin1 macrophages incubated with *Candida*-BFP. Images were taken without *Candida*-BFP or after 30 minutes, 90 minutes and 180 minutes of co-incubation with *Candida*-BFP to study phagocytosis of yeast, hyphae, and very long hyphae, respectively. White arrows indicate areas of mCherry recruitment to the *Candida*-containing phagocytic cup, visible during ingestion of *C. albicans* yeast (30 minutes) and *C. albicans* hyphae (90 and 180 minutes). (B): XYZ images of C. albicans phagocytosis by the BTK-mCherry, mCherry-Vav1 and mCherry-Syk cell lines. Cuff regions of protein recruitment are circular bands around *C. albicans* hyphae that are being ingested. (C): Quantitation of BTK-mCherry, mCherry-Vav1 and mCherry-Syk recruitment to the phagocytic cup after 90 minutes of coincubation with *Candida*-BFP. (D): Model showing localization of BTK and Vav1 to the phagocytic cup but not to mature phagosomes during phagocytosis of *C. albicans* yeast and hyphae by macrophages. Representative micrographs and means +/− SD of 3 independent experiments are shown. For statistical analysis, all data were analyzed by unpaired t test.

To study the subcellular localization of BTK, Vav1 and Syk, we constructed stable RAW macrophage cell lines that express these proteins as N- or C-terminal mCherry fusions in the RAW-Dectin1 background. In unstimulated cells, BTK-mCherry and mCherry-Vav1 were cytosolic, whereas mCherry-Syk localized to both the cytosol and nucleus (**Figure 2**, top). Next the distribution of the mCherry-tagged proteins was investigated after coincubation with *Candida*-BFP for 30, 90 or 180 min. At 30 min, when *C. albicans* yeast-form cells are present, BTK-mCherry, mCherry-Vav1 and mCherry-Syk showed a clear localization to the *Candida*-BFP-containing phagocytic cup (**Figure 2A**, arrows). After 90 and 180 min, when *C. albicans* had formed extensive hyphae, BTK-mCherry and mCherry-Vav1 showed enrichment in a cuff region of the phagocytic cup when engulfing *Candida*-BFP hyphae (**Figure 2A**, arrows). mCherry-Syk was more evenly distributed along the phagosomal membrane, with additional foci of enrichment outside the cuff region. There was no enrichment of BTK-mCherry or mCherry-Vav1 in membranes of closed, more mature, phagosomes. N- or C-terminal mCherry fusions with vimentin, expressed as controls in the same RAW macrophage cell line, did not show recruitment to the phagocytic cup (data not shown). 3D reconstructions of RAW macrophages in the process of ingesting *C. albicans* hyphae showed that the cuff regions of BTK/Vav1/Syk recruitment are cylindrical sleeves surrounding the phagosomal membrane (**Figure 2B**). Recruitment of BTK-mCherry, mCherry-Vav1 and mCherry-Syk to the phagocytic cup was quantified by comparing the fluorescence intensity in the cup/phagosomal membrane to that of the cytosol. Recruitment of mCherry-Syk was strongest, followed by mCherry-Vav1 and BTK-mCherry (**Figure 2C**).

**Figure 3:**
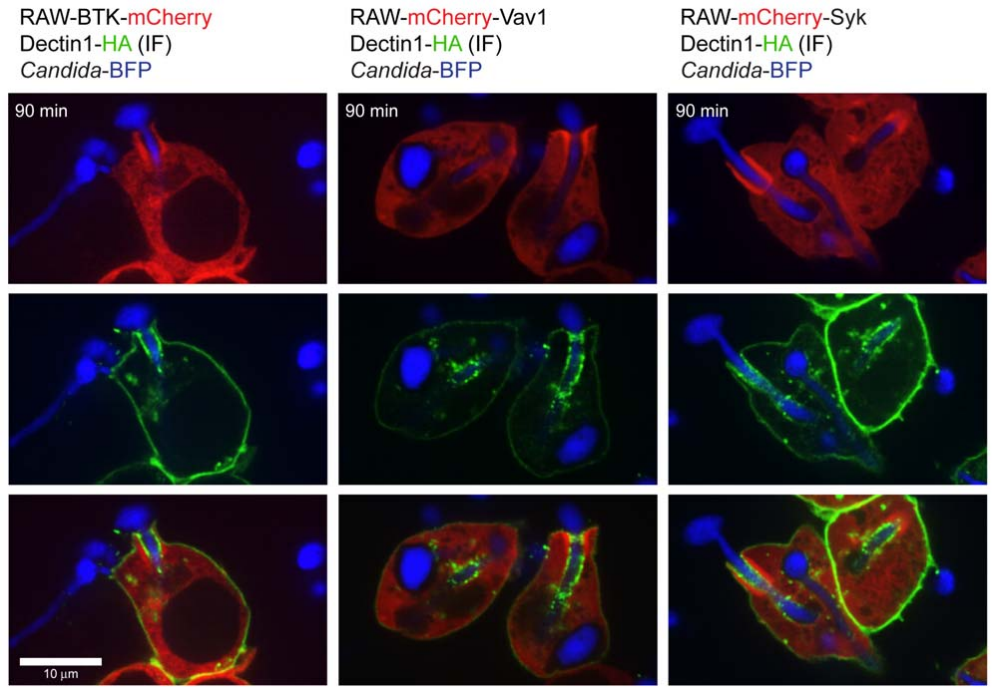
Localization of Dectin-1 during phagocytosis of *C. albicans* hyphae. Confocal images showing localization of Dectin-1 during phagocytosis of *C. albicans* hyphae by the BTK-mCherry, mCherry-Vav1 and mCherry-Syk RAW macrophage cell lines. Dectin1-HA was visualized by anti-HA immunofluorescence.

Is recruitment of BTK and Vav1 to the phagocytic cup dependent on Dectin-1? We incubated the RAW mCherry cell lines with β-glucan-coated beads or with zymosan, the phagocytosis of which is Dectin1-dependent [3]. All three fusion proteins localized to phagosomes containing β-glucan-coated beads or zymosan. Recruitment of BTK, Vav1 and Syk therefore indeed relies on Dectin-1 (**Figure S1A and B**). To address the difference in geometry of yeast versus hyphal particles, we incubated the RAW mCherry cell lines with UV-killed *C. albicans* yeasts or hyphae. While the shape of the phagocytic cup differs, recruitment of BTK, Vav1 and Syk occurs in all cases (**Figure S1C and D**). Regardless of the geometry of the ingested particle, BTK and Vav1 are recruited to the phagocytic cup but not to the mature phagosome during Dectin1-mediated phagocytosis (**Figure 2D**). Immunofluorescence was performed to investigate the localization of Dectin1 during phagocytosis of *C. albicans* hyphae. Dectin1 was enriched in the cuff region of the phagocytic cup to which BTK/Vav1/Syk were recruited, but also showed areas of enrichment outside the cuff region (**Figure 3**). Phagosomes that contain completely internalized *C. albicans* yeasts or hyphae showed very little Dectin1 staining.

### Phosphoinositide metabolism during *C. albicans* phagocytosis

Different PIs are present in the phagosomal membrane when the phagocytic cup forms, as well as during phagosomal maturation. To visualize membrane PI composition, PI-binding protein domains fused with fluorescent proteins have been used as imaging tools (biosensors or probes) [14]. We transfected the RAW-Dectin1 cell line with biosensors for PI(4,5)P_2_ (PH-PLCδ-GFP), PI(3,4,5)P_3_ (PH-BTK-GFP), PI(3,4,5)P_3_/PI(3,4)P_2_ (PH-Akt-RFP) and DAG (C1-PKCδ-GFP) to study their formation during *C. albicans* phagocytosis.

PI(4,5)P_2_ was present in the plasma membrane at rest, but multiple regions of enrichment were observed at 30 and 90 min at sites where macrophages contacted *Candida*-BFP yeast or hyphae (**Figure S2**). Membranes of sealed phagosomes no longer showed PI(4,5)P_2_ enrichment, consistent with the reported localization of PI(4,5)P_2_ during FcγR-medicated phagocytosis [10]. PI(3,4,5)P_3_ as visualized by the BTK-PH domain showed enrichment in some, but not all, of the PI(4,5)P_2_-rich regions (**Figure S2**, arrows).

**Figure 4:**
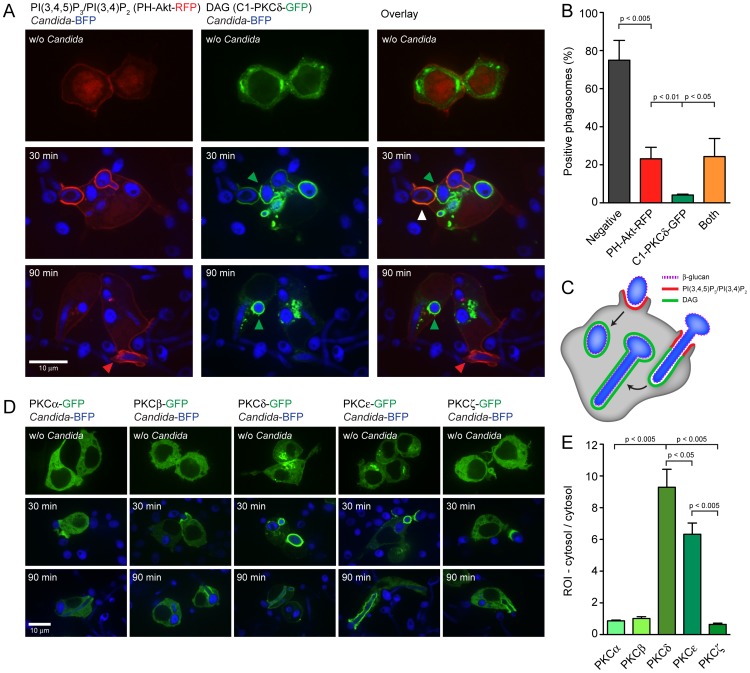
Localization of phospholipids and PKC family proteins during *C. albicans* phagocytosis. (A): PH-Akt-RFP and C1-PKCδ-GFP biosensors showing localization of PI(3,4,5)P_3_/PI(3,4)P_2_ and DAG, respectively, without challenge or after 30 or 90 minutes of co-incubation with *Candida*-BFP. White arrows indicate areas of PI(3,4,5)P_3_/PI(3,4)P_2_ and DAG co-localization, while red and green arrows indicate areas of speciation. (B): Quantitation of PI(3,4,5)P_3_/PI(3,4)P_2_- and DAG-positive phagosomes after 30 minutes of coincubation with *Candida*-BFP. (C): Model showing localization of PI(3,4,5)P_3_/PI(3,4)P_2_ and DAG during engagement and internalization of *C. albicans* yeast and hyphae by macrophages. (D): Localization of GFP-tagged PKCα, PKCβ, PKCδ, PKCε and PKCζ after 30 or 90 minutes of *Candida*-BFP phagocytosis. (E): Quantitation of PKCα-GFP, PKCβ-GFP, PKCδ-GFP, PKCε-GFP and PKCζ-GFP recruitment to the phagocytic cup after 90 minutes of coincubation with *Candida*-BFP. Representative micrographs and means +/− SD of 3 independent experiments are shown. For statistical analysis, all data were analyzed by unpaired t test.

Next we investigated the localization of PI(3,4,5)P_3_/PI(3,4)P_2_ and DAG. High levels of both PIs were present in cups or phagosomes containing *C. albicans*. PI(3,4,5)P_3_/PI(3,4)P_2_ and DAG colocalized in some early phagosomes, but phagosomes containing only PI(3,4,5)P_3_/PI(3,4)P_2_ or only DAG were also present (**Figure 4A**, white and green arrows). Recruitment of PH-Akt-RFP (PI(3,4,5)P_3_/PI(3,4)P_2_) and C1-PKCδ-GFP (DAG) or both to *Candida*-containing cups and phagosomes was quantified at the 30-min time point (**Figure 4B**). More than 20% of cups/phagosomes were PI(3,4,5)P_3_/PI(3,4)P_2_-positive and a comparable percentage was positive for both PI(3,4,5)P_3_/PI(3,4)P_2_ and DAG. At this stage 4% of cups/phagosomes were positive for DAG only. PI(3,4,5)P_3_/PI(3,4)P_2_ were more prominent in phagocytic cups, while DAG was more abundant in sealed phagosomes. These results indicate that during Dectin1-mediated phagocytosis of β-glucan-exposing *C. albicans* yeast or hyphae, PI(3,4,5)P_3_/PI(3,4)P_2_ are formed early during initiation of phagocytosis and that DAG-rich membranes/phagosomes appear at a more advanced stage (**Figure 4C**).

The presence of different PIs allows the sequential docking of a specialized set of effector proteins to the membrane during phagosomal maturation. DAG is a potent second messenger, activating members of the Protein Kinase C (PKC) family. We transfected the RAW-Dectin1 cell line with GFP-tagged PKCα, PKCβ1, PKCδ, PKCε or PKCζ to determine their localization during *C. albicans* phagocytosis. They all localized to the *C. albicans*-containing phagocytic cup (**Figure 4D**), albeit to varying degrees. The recruitment of PKCδ and PKCε was most pronounced, while the recruitment of PKCα, PKCβ and PKCζ was moderate (**Figure 4E**).

### Colocalization of BTK and Vav1 with PI(3,4,5)P but not with DAG

**Figure 5:**
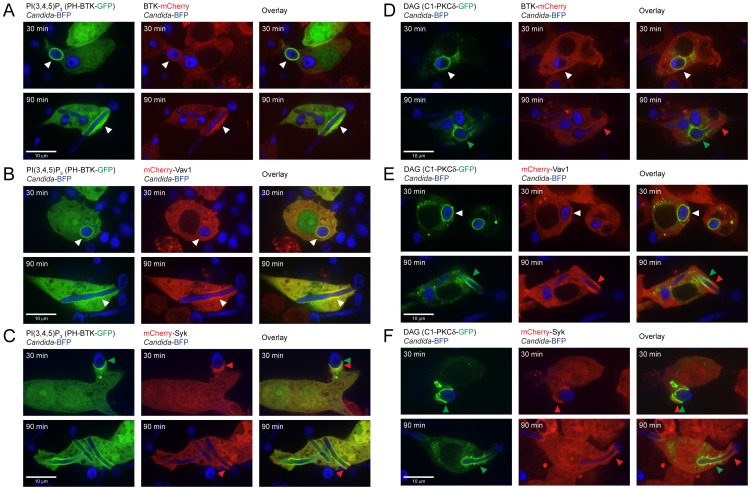
BTK-mCherry and mCherry-Vav1 colocalize with PI(3,4,5)P_3_ but not with DAG. (A): Colocalization of BTK-mCherry and PH-BTK-GFP that binds to PI(3,4,5)P_3_ at 30 and 90 minutes of coincubation with *Candida*-BFP showing phagocytosis of yeast and hyphae, respectively. (B): Colocalization of mCherry-Vav1 and PH-BTK-GFP at 30 and 90 minutes of coincubation with *Candida*-BFP. (C): Localization of mCherry-Syk and PH-BTK-GFP at 30 and 90 minutes of coincubation with *Candida*-BFP. (D): Localization of BTK-mCherry and C1-PKCδ-GFP at 30 and 90 minutes of coincubation with *Candida*-BFP. (E): Localization of mCherry-Vav1 and C1-PKCδ-GFP at 30 and 90 minutes of coincubation with *Candida*-BFP. (F): Localization of mCherry-Syk and C1-PKCδ-GFP at 30 and 90 minutes of coincubation with *Candida*-BFP. White arrows indicate areas of co-localization, while red and green arrows indicate areas of speciation. Experiments were performed at least three times, representative micrographs are shown.

BTK and Vav1 both contain a Pleckstrin Homology (PH) domain that can bind to PI(3,4,5)P_3_. Vav1 also contains a putative DAG-binding C1 domain, incapable of binding DAG owing to the presence of hydrophilic and non-charged residues in key binding positions [15]. The Syk polypeptide does not contain predicted PH or C1 domains and presumably localizes to the phagocytic cup through interaction of its tandem SH2 domains with the phosphorylated ITAM-like motif of Dectin-1. We investigated the possible colocalization of BTK, Vav1 and Syk with PI(3,4,5)P_3_ and/or DAG during phagocytosis of *C. albicans*. The BTK-mCherry, mCherry-Vav1 and mCherry-Syk RAW cell lines were transfected with the biosensor construct PH-BTK-GFP to visualize formation of PI(3,4,5)P_3_ and then incubated with *Candida*-BFP. The BTK-mCherry and mCherry-Vav1 fusion proteins colocalized with PI(3,4,5)P_3_ at the phagocytic cup, suggesting binding (**Figure 5A,B**), while recruitment of mCherry-Syk was more diffuse, confirming PI(3,4,5)P_3_-independent recruitment of Syk (**Figure 5C**, arrows). Next, we examined colocalization of BTK, Vav1 and Syk with DAG using the C1-PLCδ-GFP biosensor. BTK-mCherry showed some colocalization with DAG at 30 min. After 90 min, BTK-mCherry and DAG clearly localized to different regions of the phagocytic cup (**Figure 5D**, arrows). mCherry-Vav1 and mCherry-Syk showed a similar distribution, and neither colocalized with DAG (**Figure 5E,F**). BTK and Vav1 thus bind to PI(3,4,5)P_3_ and not to DAG during phagocytosis of *C. albicans*, consistent with the presence of PH domains in both proteins and a non-DAG binding C1 domain in Vav1. The colocalization of BTK and Vav1 with PI(3,4,5)P_3_ suggests a role for these proteins at an early stage of phagocytosis, as PI(3,4,5)P_3_ marks cups and immature phagosomes (see above).

### BTK and Vav1 localize to areas of F-actin formation at the phagocytic cup

**Figure 6:**
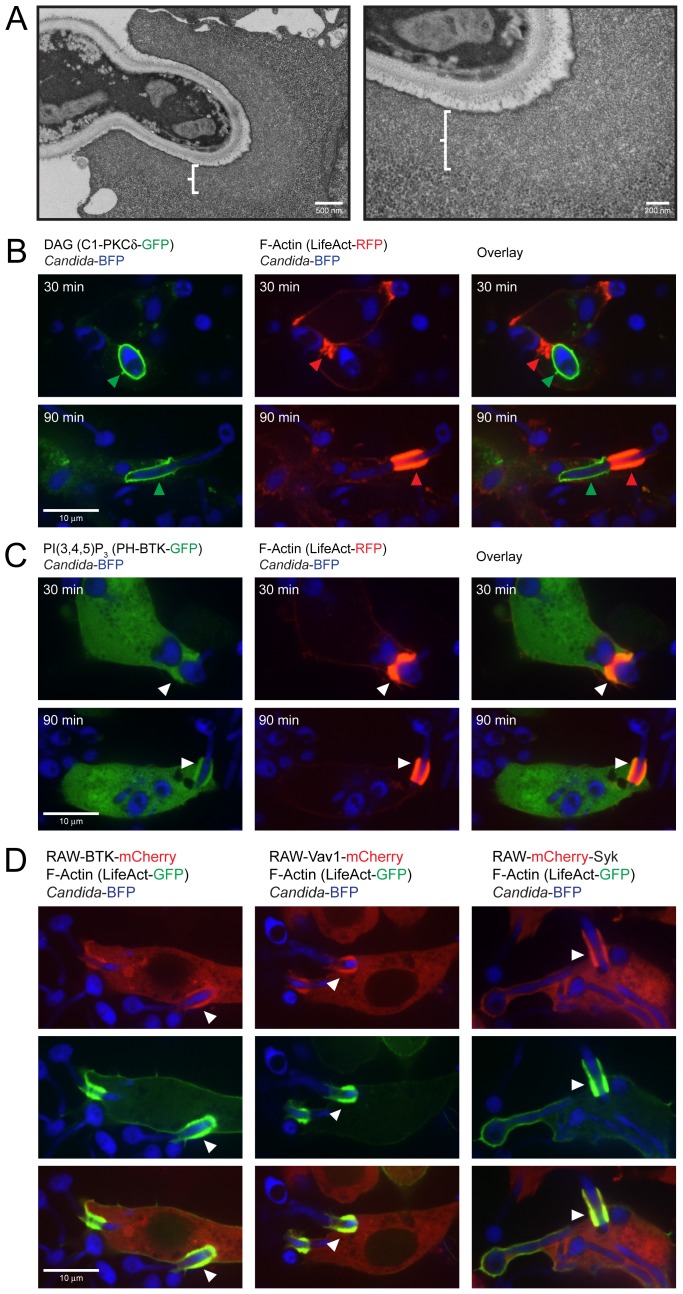
BTK, Vav1 and Syk colocalize with F-actin and PI(3,4,5)P_3_. (A): Electron microscopy of *C. albicans* phagocytosis by RAW-Dectin1 macrophages. Cells were fixed to visualize polymerized actin. (B): Localization of LifeAct-RFP detecting F-actin and C1-PKCδ-GFP that binds to DAG after 30 and 90 minutes of coincubation with *Candida*-BFP. (C): Colocalization of LifeAct-RFP detecting F-actin and PH-BTK-GFP that binds to PI(3,4,5)P_3_ after 30 and 90 minutes of coincubation with *Candida*-BFP. (D): Localization of BTK-mCherry, mCherry-Vav1 and Syk-mCherry with LifeAct-GFP after 90 minutes of coincubation with *Candida*-BFP. White arrows indicate areas of co-localization, while red and green arrows indicate areas of speciation. Experiments were performed multiple times, representative micrographs are shown.

Rearrangement of the actin cytoskeleton drives phagocytosis, enabling engulfment of the fungal particle by the macrophage. We investigated cytoskeletal changes during *Candida* phagocytosis by electron microscopy using fixation with tannic acid, a method that preserves actin structures [16]. RAW-Dectin1 macrophages were incubated with *C. albicans* for 30 min. Areas of decreased staining intensity were observed surrounding the *C. albicans*-containing phagocytic cups, indicative of actin polymerization (**Figure 6A**). These cuffs had a smooth appearance, were of uniform thickness across the sections examined and were distinct from the cytosol, which was more granular in appearance. We also examined actin polymerization with the biosensor LifeAct, which reports on the distribution of filamentous (F-) actin, in combination with biosensors that bind to PI(3,4,5)P_3_ and DAG. F-actin formation was detectable at the phagocytic cup of *Candida*-BFP yeast and hyphae at 30 and 90 min. There is a clear separation of DAG-rich regions of the phagosome from the F-actin rich membranes (**Figure 6B**). This suggests regional membrane specializations, with different functionalities and different peripheral proteins associated with them. F-actin showed perfect colocalization with PI(3,4,5)P_3_ at the *Candida*-BFP containing phagocytic cup (**Figure 6C**). Also, BTK-mCherry, mCherry-Vav1 and mCherry-Syk colocalize with F-actin after 30 and 90 min of *C. albicans* phagocytosis (**Figure 6D**). We conclude that PI(3,4,5)P_3_-rich areas are formed in the course of Dectin1-mediated phagocytosis of *C. albicans* and that BTK and Vav1 are recruited to these areas, with ensuing formation of F-actin.

### Inhibition of BTK affects phagocytosis and production of DAG

**Figure 7:**
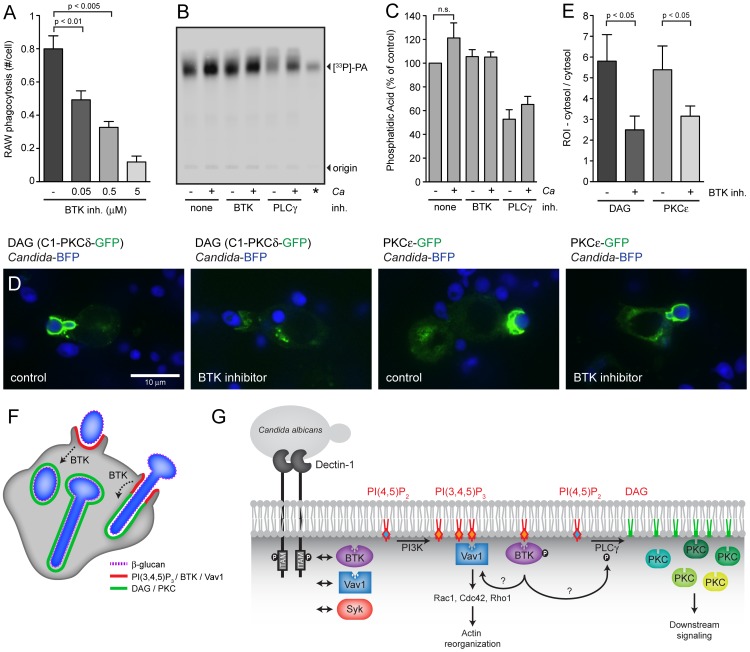
BTK is involved in DAG production at the phagocytic cup. (A): RAW-Dectin1 macrophages were pre-incubated with the indicated concentrations of BTK inhibitor PCI-32765 followed by coincubation with *Candida*-BFP (MOI 10) for 1 hour. Graphs represent number of internalized *Candida*-BFP per macrophage as determined by microscopy. (B): DAG measurements in RAW-Dectin1 macrophages preicubated with the indicated inhibitors and in the presence or absense of *C. albicans*. Thin layer chromatography was performed to visualize the phosphatidic acid (PA) product of the DAG kinase assay. (C): Quantification of PA signal from three independent DAG kinase experiments. (D): Confocal images of C1-PKCδ-GFP (DAG) and PKCε-GFP distribution in RAW-Dectin1 macrophages pre-incubated with 0.5 µM BTK inhibitor PCI-32765. (E): Quantification of C1-PKCδ-GFP and PKCε-GFP recruitment to phagosomes in absence and presence of 0.5 µM BTK inhibitor. All graphs display means +/− SD of three independent experiments. (F): Schematic showing localization of PI(3,4,5)P_3_, BTK, Vav1, DAG and PKC family proteins during engagement and internalization of *C. albicans* yeast and hyphae by macrophages. (G): Model summarizing this studies findings. BTK, Vav1 and Syk interact with Dectin-1 during phagocytosis of *C. albicans* (left). Phosphatidylinositol 4,5-biphosphate (PI(4,5)P_2_) can be converted to phosphatidylinositol 3,4,5-triphosphate (PI(3,4,5)P_3_) by PI3K or to diacylglycerol (DAG) by phospholipase C γ (PLCγ). Specialized PI(3,4,5)P_3_- and DAG-rich phagosomal membranes can be distinguished during *C. albicans* phagocytosis. Bruton's Tyrosine Kinase (BTK) and Vav1 localize to PI(3,4,5)P_3_-rich membrane regions and colocalize with F-actin. Vav1 might play an active role in actin rearrangements at the phagocytic cup through activation of small GTPases Rac1, Cdc42 and/or Rho1. BTK is involved in the production of DAG at the phagocytic cup, possibly through the activation of PLCγ. Protein Kinase C (PKC) family proteins localize to DAG-rich membranes. For statistical analysis, all data were analyzed by unpaired t test.

Having established interactions of BTK and Vav1 with Dectin-1 in the course of phagocytosis and their recruitment to PI(3,4,5)P_3_-rich membranes, we next investigated the functional importance of these proteins in phagocytosis of *C. albicans*. We synthesized the highly selective irreversible BTK inhibitor PCI-32765 [17]. The IC_50_ of this BTK inhibitor for BTK, Tec kinase and Syk is 0.5, 78 and >10,000 nM, respectively, corresponding to a BTK selectivity of 156 fold (Tec) and >10,000 fold (Syk) [17]. In B cells, PCI-32765 irreversibly inhibited autophosphorylation of BTK (IC_50_: 11 nM), phosphorylation of BTK's physiological substrate PLCγ (IC_50_: 29 nM), and phosphorylation of downstream kinase ERK (IC_50_: 13 nM) [17]. RAW-Dectin1 macrophages were pre-incubated with different concentrations of BTK inhibitor (starting from 50 nM), followed by coincubation with *Candida*-BFP for 1 hour and staining with fluorescently labeled Concanavalin A to distinguish accessible (extracellular) particles from internalized *Candida*-BPF particles. Preincubation of RAW-Dectin1 macrophages with 50 nM PCI-32765 reduced uptake of *C. albicans* by 30% and increasing inhibitor concentrations progressively blocked phagocytosis (**Figure 7A**). These results indicate an important role for BTK during *C. albicans* phagocytosis.

PLCγ, which converts PI(4,5)P_2_ into DAG, is a key enzyme during phagocytosis by innate immune cells and can be regulated by BTK [18], [19]. We hypothesized that during phagocytosis of *C. albicans*, early localization of BTK to the phagocytic cup activates PLCγ to increase synthesis of DAG and so enables recruitment of PKC members to the phagosomal membrane. To address this possibility, RAW-Dectin1 macrophages were incubated with BTK inhibitor or the PLC inhibitor U73112, followed by coincubation with *C. albicans* for 1 hour. Total DAG levels in these macrophages were measured by lipid extraction, DAG kinase assays and quantification of the product, phosphatidic acid, by thin layer chromatography. Addition of the PLC inhibitor U73112 reduced total DAG levels by 50% while the BTK inhibitor did not significantly affect total DAG levels (**Figure 7B and C**). However, in samples without inhibitor we observed a small increase in DAG levels in the presence of *C. albicans*, possibly due to increased production of DAG at the phagocytic cup during phagocytosis. To examine the effect of the BTK inhibitor on local production of DAG at the phagocytic cup, we performed confocal microscopy with the C1-PLCδ-GFP biosensor and the PKCε-GFP construct in the absence and presence of the BTK inhibitor. Addition of the inhibitor strongly reduced recruitment of the C1-PLCδ-GFP biosensor and the PKCε-GFP construct to the phagocytic cup (**Figure 7D and E**). These results underscore the importance of investigating local changes in lipid composition as opposed to changes in total levels. BTK is thus involved in the production of DAG and the subsequent recruitment of PKC family proteins at the phagocytic cup (**Figure 7F**). The interactions of BTK and Vav1 with Dectin-1, their recruitment to PI(3,4,5)P3-rich membranes, the possible contribution of Vav1/BTK to actin rearrangements and the role of BTK in the production of DAG are summarized in **Figure 7G**.

### *btk*−/− and *vav1*−/− macrophages display reduced phagocytosis

**Figure 8:**
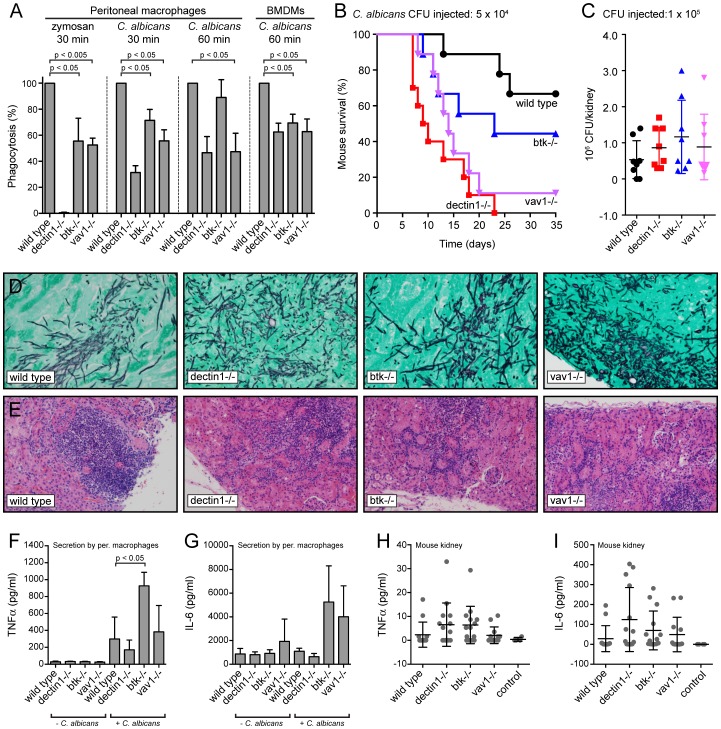
Phenotypic analysis of BTK- and Vav1-deficient macrophages and mice. (A): Peritoneal macrophages or bone marrow-derived macrophages (BMDM) from wild type, *dectin1−/−*, *btk*−/− or *vav1*−/− mice were incubated with zymosan-Alexa647 or live *Candida*-BFP for 30 minutes or 1 hour at an MOI of 10 and the number of internalized *Candida*-BFP was determined by microscopy. Graphs represent means and standard deviations of experiments with three different mice. (B): The contribution of BTK and Vav1 to overall immune responses to *C. albicans* was determined using the model for systemic candidiasis. Tail vein injection of wild type, *dectin1*−/−, *btk*−/− or *vav1*−/− mice were performed with 0.5×10^4^ colony forming units (CFU) of *C. albicans* and disease was monitored over time. (C): *C. albicans* CFU in kidneys of indicated mice at final stage of disease, means +/− SD are indicated. (D): GMS staining of kidney histology slides of wild type, *dectin1*−/−, *btk*−/− or *vav1*−/− mice, at final stage of disease showing extensive fungal invasion of tissues. (E): H&E staining of kidney histology slides of wild type, *dectin1*−/−, *btk*−/− or *vav1*−/− mice, at final stage of disease showing extensive immune cell invasion of tissues. Representative images are shown. TNFα (F) and IL-6 (G) levels in supernatant of mouse peritoneal macrophages 12 hours after incubation without or with *C. albicans*. Graphs represent means and standard deviations of experiments with three different mice. TNFα (H) and IL-6 (I) levels in kidney lysates of mice infected with 5×10^4^ CFU *C. albicans* at 11 days after infection. Each dot represents one mouse; means and standard deviations are indicated. Values did not differ significantly. For statistical analysis, all data were analyzed by unpaired t test.

To further investigate the contributions of BTK and Vav1 to phagocytosis we determined the phagocytic capacity of peritoneal macrophages and bone marrow-derived macrophages (BMDMs) from wild type mice and from *dectin-1*, *btk* and *vav1* knockout mice. Phagocytic indices were determined after incubation of peritoneal macrophages with zymosan for 30 min or with *C. albicans* for 30 min or 1 hour. While Dectin1-deficient peritoneal macrophages did not phagocytose zymosan, the *btk* and *vav1* deficient cells showed an intermediate phenotype (**Figure 8A**). Uptake of *C. albicans* by *btk* and *vav1* knockout peritoneal macrophages at the 30-min time point was also significantly reduced compared to wild type cells (p<0.05). In addition, the *btk−/−* and *vav1*−/− BMDMs displayed a reduction in phagocytosis similar to that seen for the *dectin1−/−* BMDMs (p<0.05) (**Figure 8A**). BTK and Vav1 are thus important contributors to Dectin1-mediated uptake of *C. albicans* by BMDMs.

### *btk−/−* and *vav1*−/− mice show increased sensitivity to *C. albicans* infections

*In vivo* immune responses of wild type and *dectin1−/−*, *btk−/−* and *vav1*−/− mice to *C. albicans* were tested in a systemic candidiasis model. Tail vein injection of the four groups of mice with *C. albicans* showed that *dectin1*−/− mice were most susceptible to *C. albicans* infections, while the majority of the wild type animals survived the systemic infection. *Btk−/−* and *vav1*−/− animals displayed an intermediate phenotype, both during systemic infection with 5×10^4^ colony forming units (CFU) (**Figure 8B**) and with 1×10^5^ colony forming units (CFU) of *C. albicans* (data not shown). We found no statistical difference in *C. albicans* loads in kidneys harvested from mice about to succumb to infection (**Figure 8C**). In addition, histological analysis of the kidneys showed similar fungal loads (**Figure 8D**) and comparable immune cell invasion of the tissues in the different groups (**Figure 8E**). Elevated chemokine and cytokine levels in the kidney represent early responses to *C. albicans* infection and correlate with virulence [20]. We determined secretion of the proinflammatory cytokines TNFα and IL-6. Wild type, *dectin1−/−*, *btk−/−* and *vav1−/−* peritoneal macrophages secreted both TNFα and IL-6 (**Figure 8F,G**) in response to exposure to *C. albicans. Btk−/−* and *vav1−/−* macrophages generally secreted more TNFα and IL-6 than wild type and *dectin1−/−* macrophages (the difference between wild type and *btk−/−* TNFα secretion reached statistical significance). Next we investigated TNFα and IL-6 levels in mouse kidney during systemic *C. albicans* infection. Kidneys were harvested 11 days after tail vein injection with 5×10^4^ CFU. Although levels of TNFα and IL-6 were slightly higher in the *dectin1−/−*, *btk−/−* and *vav1−/−* mice than in wild type, these differences did not reach statistical significance (**Figure 8H,I**). We conclude that disease progression in response to *C. albicans* systemic infection is accelerated in *dectin1−/−*, *btk−/−* and *vav1*−/− animals compared to wild type animals.

## Discussion

BTK and Vav1 are best known for their role in adaptive immunity: BCR signaling in B cells (BTK) and B and T cell development as well as activation of mature lymphocytes (Vav). Vav family members orchestrate cytoskeletal rearrangements, with Vav1 being expressed in the hematopoietic system in particular [21]. BTK and Vav also participate in innate immune reactions. BTK contributes to Fc-mediated phagocytosis [22] and was previously implicated in Dectin1-dependent pathways [23], [24], while the Vav protein family participates in Dectin-1/Mac-1 signaling in neutrophils [25]. We identified BTK and Vav1 as novel interaction partners of the β-glucan receptor Dectin-1 and confirmed their importance during *C. albicans* phagocytosis and immune responses during systemic infection with *C. albicans*. Dectin-1/BTK and Dectin-1/Vav1 complexes form during phagocytosis of live *C. albicans*, particularly during ingestion of *C. albicans* hyphae (**Figure 1** **and** **2**). BTK and Vav1 are found at membranes enriched for PI(3,4,5)P_3_ and colocalize with markers for F-actin (**Figures 5** **and** **6**) where BTK is involved in production of DAG at the phagocytic cup (**Figure 7**). Macrophages deficient in BTK or Vav1 display reduced phagocytosis and BTK- or Vav1-deficient mice succumb more readily to systemic *C. albicans* infections than do wild type animals (**Figure 8**).

BTK and Vav1 can now be added to the list of Dectin1-interacting proteins, which includes Syk [4], PKCδ [5], the tetraspanin CD37 [6], Galectin-3 [7] and TLR2 [8]. The multiple interactions of Dectin-1 with its partners reflect the complexity of Dectin-1 signaling and the (sub)complexes in which it participates. Under non-phagocytic conditions, the receptor remains at the plasma membrane, but engagement by a β-glucan ligand initiates phagocytosis and signaling from the nascent phagosome. With multiple Dectin1-interacting proteins identified, the interesting possibility of tripartite or multicomponent signalosomes arises. Dectin-1 multicomponent signalosomes exist, as complexes were found that encompassed PKCδ, Syk and Dectin-1 [5]. We tested the possibility of a Dectin1/BTK/Vav1 tripartite complex, but could not detect the three proteins in a single complex (data not shown). However, Vav1 could be a target of BTK phosphorylation, as the SH3 domain of BTK interacts with Vav1 in B cells [26]. The signaling events that occur after engagement of Dectin-1 and the relationships between BTK, Vav1 and the known Dectin-1 mediator Syk remain to be clarified. Although interaction and signaling data cannot be extrapolated to different cell types, relevant information can be extracted from the literature. Syk and Vav interact in yeast-two-hybrid experiments and in B and T cells, where Syk directly phosphorylates Vav [27].

BTK and Vav1 localize to the *Candida*-containing phagocytic cup, both when *C. albicans* yeasts and hyphae are internalized (**Figure 2**). During phagocytosis of hyphae, BTK-mCherry and mCherry-Vav1 show strong recruitment to a “cuff” region where engulfment of the hyphae is ongoing. *C. albicans* hyphae grown under these conditions expose high levels of β-glucan (**Figure 1**). The strong RAW-Dectin1 engagement in the cuff regions and the ensuing recruitment of BTK and Vav1 could therefore be a result of increased exposure of β-glucan on the *C. albicans* hyphae under these conditions. The exposure of β-glucan on *C. albicans* yeast and hyphae is a matter of ongoing debate.

Our data show that hyphae generated by growth in DMEM media with serum at 37°C for 90–180 min display high levels of β-glucan (Figure 1A), which is also the case during disseminated infection [28]. However, it was also reported that Dectin1 does not bind to *C. albicans* hyphae generated by overnight growth at 37°C in serum-free RPMI media [29], producing hyphae that appear morphologically distinct. Different growth conditions may well produce differences in β-glucan exposure and yield distinct hyphal morphologies.

Phosphoinositides (PIs) formed in the phagosomal membrane serve as docking stations for proteins with the appropriate binding domains. During phagocytosis of *C. albicans*, PI(4,5)P_2_ is enriched at sites of contact, followed by production of PI(3,4,5)P_3_ at the phagocytic cup and disappearance of PI(4,5)P_2_ and DAG-enrichment as the phagosomes seal (**Figures S2 and** **Figure 3**). BTK and Vav1 colocalize with PI(3,4,5)P_3_ (**Figure 5**), which suggests a role for these proteins at an early stage of phagocytosis. F-actin also colocalizes with BTK and Vav1 in the PI(3,4,5)P_3_-rich areas, which fits the known role of Vav1 in actin cytoskeleton rearrangement and suggests a possible contribution of BTK to F-actin formation (**Figure 6**). The observation that Vav1 colocalizes with PI(3,4,5)P_3_ but not with DAG supports the notion that the C1 domain of Vav1 is not a functional DAG-binding domain [15]. During CR3- and FcγR-mediated phagocytosis, actin tail formation follows local production of PI(3,4,5)P_3_ [30]. BTK also localizes to actin-rich cups during FcγR-mediated phagocytosis [22]. Our data and those in the literature emphasize similarities between Dectin1- and FcγR-mediated phagocytosis.

The enzyme PLCγ converts PI(4,5)P_2_ to DAG at the phagocytic cup and the PLC inhibitor U73112 blocked *C. albicans* phagocytosis by the RAW-Dectin1 macrophages (data not shown). PLCγ is also essential for FcγR-mediated phagocytosis [10]. DAG-rich membranes recruit proteins of the PKC family that have a DAG-interacting C1 domain [31], [32]. All PKC isoforms examined localized to the *Candida*-containing phagocytic cup, with the Ca^2+^-independent family members PKCδ and PKCε displaying the strongest recruitment (**Figure 3**). Conventional PKCs require increased intracellular Ca^2+^ levels for activation and binding to DAG [33] but DAG-independent recruitment of the different PKC isoforms, for example via protein-protein interactions, remains possible as well. PKCδ and PKCε are involved in early steps of phagocytosis: PKCδ interacts with Dectin-1 and Syk and is required for phagocytosis of zymosan [5] whereas PKCε enhances FcγR-mediated phagocytosis [34]. Conventional PKCs also contribute to other processes, such as the generation of the respiratory burst, but are dispensable for FcγR-mediated internalization [35]. Further characterization of the contributions of individual PKC members to FcγR- and Dectin1-mediated phagocytosis is necessary.

BTK and Vav1 are important for phagocytosis of *C. albicans*, as *btk−/−* and *vav1−/−* peritoneal macrophages and BMDMs displayed reduced phagocytosis (**Figure 8**). BTK- and Vav1-deficient mice are also more susceptible to systemic infections with *C. albicans*. Dectin1-, BTK- and Vav1-deficient mice succumb earlier to infections than do wild type mice, but fungal burdens, immune cell invasion and cytokine levels in the kidney are comparable (**Figure 8**). While the phenotype of the BTK- and Vav1-deficient mice might be due to reduced phagocytosis by macrophages, the mice used here are complete knockouts. We therefore must remain vigilant to the possibility that defects in macrophage or innate immune cells may not be solely responsible for the results reported here. *Btk−/−* mice have B cell defects [36], [37] while *vav1−/−* mice have reduced numbers of T and B cells [38], [39]. Although B and T cells are not thought to play a major role in immune responses during systemic *C. albicans* infections, a (minor) contribution cannot be excluded. In addition to their role in phagocytosis, BTK and Vav1 might contribute to other innate processes related to *C. albicans* immune responses, such as production of reactive oxygen species or cytokines. The complexities of these interconnections clearly require further study.

BTK and PLCγ are functionally connected, as knockdown of BTK resulted in reduced PLCγ phosphorylation in response to stimulation of the TREM-1/DAP12 pathway in a lymphoma cell line [19]. In our RAW-Dectin1 cell line, pharmacological inhibition of BTK resulted in decreased phagocytosis, reduced DAG levels and compromised recruitment of PKCε to the phagocytic cup (**Figure 7**). We therefore hypothesize that in this setting BTK is responsible for activation of the DAG-producing enzyme PLCγ. In FcγR-mediated phagocytosis, PLCγ phosphorylation and recruitment to the phagocytic cup are Syk-dependent [10], [40]. It remains to be established if Syk or BTK or both are responsible for PLCγ phosphorylation and/or activation during Dectin1-mediated phagocytosis. BTK was previously shown to be involved in Dectin1-dependent arachidonate release by macrophages in response to incubation with zymosan or particulate β-glucan [23], [24]. Phosphorylation of BTK on tyrosine 223 and phosphorylations of PLCγ2 were induced by incubation with zymosan or particulate β-glucan. However, incubation with the BTK inhibitor LFM-A13 did not reduce phosphorylation of PLCγ2 during incubation with zymosan [24].

The role for Vav proteins remains incompletely understood. Our data add to previous observations that Vav proteins are instrumental in phagocytosis, and participate in a cell type-dependent manner. *vav1*/*vav2/vav3* triple-knockout macrophages ingest IgG-opsonized erythrocytes normally [41] but *vav1−/−* and *vav3*−/− neutrophils are deficient in FcγR-mediated phagocytosis [42]. With regards to fungal particles, in microglia phosphorylation of Vav1 is induced by particulate β-glucan and this phosphorylation is affected by a Src family kinase inhibitor as well as a Syk inhibitor. Vav1 knockdown in a microglial cell line resulted in reduced uptake of β-glucan particles [43]. *vav1*/*vav3* double knockout neutrophils show reduced binding to zymosan, and *vav1*/*vav3* mice have increased susceptibility to *C. albicans* infection [25] accompanied by reduced PLCγ phosphorylation. While in our hands *vav1* peritoneal macrophages have defects in both *C. albicans* and zymosan uptake (**Figure 8A**), Li et al. reported that thioglycollate-induced peritoneal macrophages do not display reduced phagocytosis of zymosan [25]. Whether these differences are due to macrophage activation status remains to be established. In addition, the precise role of Vav1 and a possible link to PLCγ during phagocytosis by macrophages remains incompletely understood.

Differences in cell wall composition between *C. albicans* and a non-pathogenic yeast like *Saccharomyces cerevisiae* might influence recruitment of downstream factors. A systematic assessment of the involvement of BTK, PLCγ, DAG and PKC family members during Dectin1-mediated phagocytosis of *C. albicans, S. cerevisiae* and other fungi should help clarify their roles.

## Materials and Methods

### Ethics statement

Animals used in this study were housed at the Whitehead Institute for Biomedical Research, which is certified by the United States Office of Laboratory Animal Welfare (OLAW) under the guidance of the Public Health Service (PHS) Policy on Humane Care and Use of Laboratory Animals. Whitehead Institute's Animal Welfare Assurance was approved 11/3/2009 (IACUC, A3125-01) All studies were carried out in accordance with procedures approved by the Massachusetts Institute of Technology Committee on Animal Care (Ploegh lab, CAC# 1011-123-14).

### Cells and culture conditions

*C. albicans* strain SC5314 was cultured in YPD + Uri (2% bactopeptone, 1% yeast extract, 2% glucose and 80 µg/ml uridine) at 30°C. To generate a blue fluorescent protein (BFP)-expressing *C. albicans* strain, the GFP sequence of the pENO1-yEGFP3-NAT plasmid [28] was replaced with the TagBFP sequence (Evrogen) with codon usage adapted for *C. albicans*. *C. albicans* SC5314 was transformed with the pENO1-TagBFP-NAT plasmid and selected with 200 µg/ml nourseothricin (Werner Bioagents, Jena, Germany) resulting in the *Candida*-BFP strain. The RAW-Dectin1-LPETG-3×HA cell line (RAW-Dectin1) [7] was used for most phagocytosis experiments. Cells were grown in DMEM medium with 10% inactivated Fetal Bovine Serum (IFS) at 37°C and 5% CO_2_. For the production of retrovirus, HEK293T cells (ATCC) were transfected using TransIT transfection reagent (Mirus) and virus-containing supernatant was harvested after 24 hours.

### Plasmids, cloning, protein expression and construction of stable cell lines

The imaging constructs used in this study are listed in Table 1. A Dectin1-CRD-LPETG-His bacterial expression vector was cloned and expressed as described for Dectin1-CRD [13]. Mammalian expression vectors were constructed for N- or C-terminal mCherry fusions in vectors based on the retroviral plasmid pMSCVpuro (Clontech) (pMSCVpuro-mCherry-N and pMSCVpuro-mCherry-C). The BTK, Vav1 and Syk open reading frames were cloned from mouse spleen cDNA into the tagging vectors, resulting in N- or C-terminal fusion of all three genes (mCherry-BTK, BTK-mCherry, mCherry-Vav1, Vav1-mCherry, mCherry-Syk and Syk-mCherry). The vectors were used to create stable cell lines in the RAW-Dectin1 background by retroviral transduction and selection with puromycine. The resulting six cell lines were tested for expression of the mCherry-fused proteins by immunoblotting and microscopy and the BTK-mCherry, mCherry-Vav1 and mCherry-Syk cell lines were selected for further experiments. For expression of the biosensor constructs in the RAW macrophage cell lines, cells were transfected using Fugene (Roche).

**Table 1:** Imaging constructs used in this study.

Plasmid	Detects	Abbreviation	Reference
PH-PLCδ-RFP	Phosphatidylinositol 4,5-bisphosphate	PI(4,5)P_2_	[50]
PH-Akt-RFP	Phosphatidylinositol 3,4,5-trisphosphate and phosphatidylinositol 3,4-bisphosphate	PI(3,4,5)P_3_ and PI(3,4)P_2_	[51]
PH-BTK-GFP	Phosphatidylinositol 3,4,5-trisphosphate	PI(3,4,5)P_3_	[52]
C1-PKCδ-GFP	Diacylglycerol	DAG	[53]
LifeAct-RFP	Filamentous actin	F-actin	[54]
LifeAct-GFP	Filamentous actin	F-actin	[54]
PKCα-GFP	Protein Kinase C isoform α	PKCα	[55]
PKCβ-GFP	Protein Kinase C isoform β	PKCβ	[55]
PKCδ-GFP	Protein Kinase C isoform δ	PKCδ	[55]
PKCε-GFP	Protein Kinase C isoform ε	PKCε	[55]
PKCζ-GFP	Protein Kinase C isoform ζ	PKCζ	[55]
BTK-mCherry	Bruton's Tyrosine Kinase	BTK	This study
mCherry-Vav1	Guanine nucleotide exchange factor Vav1	Vav1	This study
mCherry-Syk	Spleen Tyrosine Kinase	Syk	This study

### Immunoprecipitation, mass spectrometry, blotting and antibodies

To identify proteins that interact with Dectin-1 during phagocytosis, RAW-Dectin1 macrophages were incubated with live *C. albicans* at MOI 5 for 1 hour or left unchallenged. Cells were harvested by scraping into ice-cold PBS and lysed in NP40 buffer (25 mM Tris pH 7.4, 150 mM NaCl, 5 mM MgCl_2_ with 0.5% NP40 and protease inhibitors). Epitope-tagged Dectin-1 was immunoprecipitated from the total lysates with anti-HA beads (Roche). Eluates were run on a SDS-PAGE gradient gel and silver stained to visualize proteins. Each lane was cut into regions according to molecular weight, which were then reduced, alkylated and subjected to trypsin digestion. The resulting peptides were extracted, concentrated *in vacuo*, and analyzed by reverse-phase chromatography and tandem mass spectrometry. The resulting CID spectra were searched against a species-specific database generated from NCBI's non-redundant database using SEQUEST. For the generation of anti-BTK and anti-Vav1 antisera, BTK and Vav1 were cloned from mouse spleen cDNA into bacterial expression vector pET28a with an N-terminal His tag (pET28a-BTK and pET28a-Vav1). Vectors pET28a-BTK and pET28a-Vav1 were used to transform Rosetta cells and transformants were induced with IPTG for protein expression. His-Vav1 was isolated from the soluble fraction and His-BTK was isolated from the insoluble fraction in 8 M urea. Both proteins were purified using NiNTA beads and BTK was refolded by stepwise dialysis to eliminate urea, followed by FPLC purification of the peak containing the monovalent BTK. Purified Vav1 and BTK were injected in rabbits and serum was harvested and used for immunoblot and immunoprecipitation experiments (anti-BTK and anti-Vav1). Other antibodies used were: anti-Syk (Cell Signaling), anti-phospho-Syk (Tyr525/526; Cell Signaling), anti-PLCγ2 (Cell Signaling), anti-phospho-PLCγ2 (Tyr1217; Cell Signaling), anti-p97 [44], anti-HA-HRP (Roche). For immunoblotting, protein extracts were separated on 8% or 12% SDS-polyacrylamide gels and transferred to a nitrocellulose membrane using a semi-dry system. For BTK and Vav1 immunoprecipitation experiments, cells were lysed in NP40 buffer followed by immunoprecipitation with 2 µl of anti-BTK or anti-Vav1 antisera and 30 µl of Protein-A beads (Repligen). Beads were washed and eluates were analyzed using SDS-PAGE gels.

### Spinning disk confocal and electron microscopy

Confocal images were collected in the W.M. Keck Facility for Biological Imaging using a PerkinElmer Live Cell Imaging spinning disk confocal system and Volocity software. The PerkinElmer Live Cell Imaging spinning disk confocal system was mounted on a Zeiss Axiovert 200M with a 100× 1.4NA Plan-Apochromat objective. Excitation light was generated by gas and solid state lasers (Argon laser for 488 nm, Krypton laser for 568 nm, solid state laser for 405 nm and 647 nm) and passed through an AOTF for wavelength selection and laser power control. A quadruple bandpass filter separated the excitation and emission light inside the CSU-22 confocal scanhead (Yokogawa) and a filter wheel (Prior Scientific) provided selection of emission filters (TagBFP & RFP: dual-band 445/60 and 615/70 nm; GFP: 527/55 nm). Volocity image acquisition software was used to capture images from a Hamamatsu Orca-ER cooled-CCD camera and to control all the equipment. For 3D reconstructions of phagocytic cells, Z planes were acquired at 0.15 µM distance and Volocity image acquisition software was used to create the XYZ views. Electron microscopy sections were examined using a FEI Tecnai Spirit at 80 KV. Routine morphology was performed by trimming and fixing the tissue in 2.5% gluteraldehyde, 3% paraformaldehyde with 5% sucrose in 0.1 M sodium cacodylate buffer (pH 7.4) and 0.2% tannic acid. Samples were post fixed in 1% osmium in veronal-acetate buffer. The tissue was stained in block overnight with 0.5% uranyl acetate in veronal-acetate buffer (pH 6.0), then dehydrated and embedded in em812 resin. Sections were cut on a Leica Ultracut UCT microtome with a Diatome diamond knife at a thickness setting of 50 nm, stained with uranyl acetate, and lead citrate. The sections were examined using a FEI Tecnai Spirit at 80 KV.

### Phagocytosis assays

For general confocal microscopy, *Candida*-BFP was added to RAW-Dectin1 macrophages at an MOI of 10, fixed with 4% PFA in PBS and mounted on slides in 50% glycerol. β-1,3-glucan conjugated beads were a kind gift of Jatin Vyas and prepared as described [45]. Zymosan A (Sigma) was labeled with Alexa647 carboxylic acid, succinimidyl ester (Invitrogen) by incubation in 0.1 M Na_2_CO_3_ at room temperature. *Candida*-BFP was UV-killed by exposure to 100.000 µJ/cm^2^ in a UV-crosslinker for four rounds. Recruitment of fluorescent proteins to the phagocytic cup or phagosome was quantified using ImageJ software according to the method of Flannagan and Grinstein [46]. Phagocytic indices of RAW-Dectin1 cells, BMDMs or peritoneal macrophages were determined by incubation with *Candida*-BFP or zymosan-Alexa647 at MOI 10 for 30 minutes or 1 hour. Cells were fixed in 4% PFA and stained with Concanavalin A-FITC (Sigma) to distinguish unengulfed yeasts. Inhibitors were added to the media at the indicated concentrations for 1 hour prior to incubation with *Candida*-BFP. Images were aquired by confocal microscopy and the number of intracellular *Candida*-BFP per macrophage was determined by counting 75-200 cells per experiment. Dectin1-CRD-LPETG was incubated with *Staphylococcus aureus* sortase A enzyme and GGG-Alexa647 probe resulting in Dectin1-CRD-LPETGGG-Alexa647 that was used for staining of *Candida*-BFP yeasts and hyphae. For immunofluorescence, cells were grown on coverslips and fixed in 4% PFA in PBS, washed with PBS and incubated in 50 mM NH_4_CL in PBS for 10 min. Next, cells were incubated in Binding Buffer (0.1% Saponin, 0.2% BSA in PBS) for 30 min followed by incubation in Binding Buffer with anti-HA-Alexa488 (Invitrogen) antibody for 60 min, several washes with PBS and mounting for spinning disk confocal microscopy.

### Synthesis of BTK inhibitor PCI-32765

4-Amino-3-(4-phenoxyphenyl)-1H-pyrazolo[3,4-d]pyrimidine (**1**) was prepared from 4-phenoxybenzoic acid and malonitrile as described (International Patent Publication No. WO 01/019829 and [17]. Alkylation of pyrazole (**1**) with 3-methanesulfonyl N-Boc hydroxypiperidine (**2**) followed by removal of the Boc-protecting group and acylation with acryloyl chloride gave the racemate of **PCI-32756** in three steps (**Figure S3**). All chemicals were of commercial sources and were used as received. DriSolv anhydrous CH2Cl2, DriSolv anhydrous MeOH, DriSolv anhydrous DMF were purchased from EMD Chemicals. Redistilled, anhydrous *N,N′*- diisopropylethylamine (DiPEA), trifluoroacetic acid (TFA), triisopropylsilane (TIS) *N*-methylpyrrolidone (NMP) was obtained from Sigma-Aldrich. LC-ESI-MS analysis was performed using a Micromass LCT mass spectrometer (Micromass MS Technologies, USA) and a Paradigm MG4 HPLC system equipped with a HTC PAL autosampler (Michrom BioResources, USA) and a Waters Symmetry 5 µm C8 column (2.1×50 mm, MeCN∶H_2_O (0.1% formic acid) gradient mobile phase, 150 µL/min). HPLC purifications were achieved using an Agilent 1100 Series HPLC system equipped with a Waters Delta Pak 15 µm, 100 Å C18 column (7.8×300 mm) using A: H_2_O, B: MeCN and C: 1% aqueous trifluoroacetic acid as mobile phase (3 mL/min). (R/S)-1-Boc-3-Hydroxypiperidine (1.05 g, 5 mmol) was dissolved in CH_2_Cl_2_ (25 mL) and subsequently triethylamine (1.39 mL, 10 mmol) and methanesulfonyl chloride (0.394 mL, 5.1 mmol) were added. After stirring overnight, the reaction was concentrated under reduced pressure, redissolved in ethyl acetate, washed with water and brine, dried over MgSO_4_ and concentrated *in vacuo*. The crude mesylate was dissolved in anhydrous DMF (20 mL). Pyrazole **1** (1.02 g, 3.33 mmol) and potassium carbonate (0.92 g, 6.66 mmol) were added and the reaction was stirred until TLC analysis showed complete conversion. The reaction was diluted with water and extracted with CH_2_Cl_2_. The organic layer was dried over MgSO_4_, concentrated *in vacuo*. Purification over silica gel chromatography (CH_2_Cl_2_→MeOH/CH_2_Cl_2_) gave intermediate **3**. Intermediate **3** was dissolved in dioxane (20 mL) and freshly prepared hydrochloric acid (35 mmol) was added. The solution was stirred for 1 h, concentrated *in vauo*. The crude amine (1 g, 2.58 mmol) was redissolved in CH_2_Cl_2_ (10 mL). To this was added Et_3_N (1.8 mL, 12.9 mmol) and acryloyl chloride (0.22 mL, 2.71 mmol). After 5 h, the reaction was quenched by the addition of water. The solution was extracted and the organic layer was dried over MgSO_4_ and concentrated *in vacuo*. The crude product was purified by reverse phase HPLC (28→43%B in 20 min, 3 mL/min) affording the title compound (46.3 mg, 0.105 mmol) as a white solid. LC/MS: R_t_ 8.52 min; linear gradient 5→80% B in 10 min; ESI/MS: *m/z* = 441.2 [M+H]^+^. ^1^H NMR (400 MHz, CD_3_OD) δ ppm 8.39 (s, 1H), 7.69-7.66 (m, 2H), 7.44-7.39 (m, 2H), 7.21-7.08 (m, 5H), 6.82 (dd, *J* = 16.8, 10.8 Hz, 0.6H), 6.66 (dd, *J* = 16.8, 10.8 Hz, 0.4H), 6.17 (d, *J* = 16.8 Hz, 0.6H), 6.15 (d, *J* = 16.8 Hz, 0.4H) 5.77 (d, *J* = 10.8 Hz, 0.6H), 5.65 (d, *J* = 10.8 Hz, 0.4H), 4.95-4.93 (m, 1H), 4.56 (d, *J* = 12.4 Hz, 0.6H), 4.24 (d, *J* = 11.2 Hz, 1H), 4.06 (d, *J* = 14.0 Hz, 0.6H), 3.89 (dd, *J* = 12.8, 8.8 Hz, 0.4H), 3.58 (dd, *J* = 12.4, 9.6 Hz, 0.6H), 3.42-3.36 (m, 1H), 2.45-2.34 (m, 1H), 2.28-2.23 (m, 1H), 2.15-2.05 (m, 1H), 1.80-1.68 (m, 1H). 2.72 (t, *J* = 7.6 Hz, 2H), 2.15 (dt, *J* = 7.2, 2.8 Hz, 2H), 1.96 (d, *J* = 2.8 Hz, 1H), 1.82 (quin., *J* = 7.6 Hz, 2H), 1.52 (quin., *J* = 7.2 Hz, 2H).

### Quantitative analysis of cellular diacylglycerol content

RAW-Dectin1 macrophages were coincubated with *Candida albicans* in 6-well dishes for 1 hour, washed with phosphate-buffered saline and subjected to lipid extraction [47]. The chloroform/methanol phase was dried under N_2_ and DAG kinase assays were performed as described [48]. See supplementary materials for further details. The dried lipids were dissolved in 40 µl of solubilizing buffer (7.5% octyl-ß-D-glucoside and 5 mM cardiolipin in 1 mM diethylenetriaminepenta acetic acid (DETAPAC, pH 7.0) by vigorously vortexing for 20 sec and incubating at RT for 10 min. Then, 100 µl of 2× reaction buffer (100 mM imidazole HC1, pH 6.6, 100 mM NaCl, 25 mM MgCl_2_, and 2 mM EGTA), 4 µl of 100 mM freshly prepared DTT and 20 µl of *E. coli* DAG kinase (Sigma-Aldrich)) were added while keeping the samples on ice. The reaction was initiated by addition of 3 µCi [γ^33^P]-ATP prepared by dilution in 20 µl of 1 mM DETAPAC, pH 6.6. After vortexing briefly, the reaction was incubated at 25°C for 30 min. Lipids were extracted as described above and the reaction products were analyzed by TLC, which was developed in acetone followed by CHCl_3_/MeOH/acetic acid (65/15/5 [vol/vol/vol]) solution. Radiolabelled lipids were detected by exposure to imaging screens (BAS-MS; FujiFilm), scanned on a BAS-2500 (FujiFilm)), and quantified with Quantity One software.

### Animal care, primary cells and mouse model for systemic candidiasis

Animals were housed at the Whitehead Institute for Biomedical Research and maintained according to protocols approved by the Massachusetts Institute of Technology Committee on Animal Care. C57BL/6 mice were purchased from Jackson Labs, *dectin1−/−* [49], *btk−/−* [36] and *vav1−/−* mice [39] were kind gifts from Stu Levitz, Whasif Khan and Victor Tybulewicz, respectively. Bone marrow-derived macrophages (BMDMs) were differentiated from mouse bone marrow by growth in DMEM (high glucose; Gibco) with 10% HI-FBS (Hyclone) and 5% M-CSF-containing culture supernatant from L929 cells. Experiments were performed after 7 days of differentiation. Peritoneal macrophages were harvested by peritoneal lavage with upto 10 ml PBS. Cells were seeded for experiments in DMEM (high glucose; Gibco) with 10% HI-FBS (Hyclone) and used for experiments the next day. For cytokine analysis, macrophages were incubated with *C. albicans* and supernatants were harvested after 16 hours. Cytokine concentrations were determined by Discovery assay cytokine array by Eve Technologies. For the mouse model of systemic candidiasis, 5×10^4^ or 1×10^5^ CFU of *C. albicans* SC5314-derived strain *Candida*-BFP was administered intraveneously to age-matched C57BL/6 wild type, *dectin-1*, *btk* and *vav1* −/− mice in a final volume of 200 µl in PBS. Mice were weighed and monitored daily and euthanized when >20% of initial body weight was lost. Kidneys were harvested for enumeration of fungal burden, histology or cytokine analysis. To determine fungal burden, kidneys were homogenized and lysates were plated on YPD plates with antibiotics. For histology, tissues were fixed in 4% formalin in PBS, embedded and sectioned in paraffin and slides were stained with Hematoxylin and Eosin (H&E) or Gomori Methenamine Silver (GMS). For cytokine analysis, kidneys were homogenized in 1 ml PBS followed by cytokine analysis using a Th1/Th2/Th17 mouse cytometric bead array (BD Biosciences) and LSR Flow Cytometer according to the manufacturer's directions.

### Statistical analysis

For statistical analysis unpaired t tests were used with 95% confidence interval. All graphs show means and standard deviations of three independent experiments.

### Supplemental information

Supplemental information includes three figures.

## Supporting Information

Figure S1 **Localization of BTK-mCherry and Vav1-mCherry to the phagocytic cup.** Confocal images showing localization of BTK-mCherry, mCherry-Vav1 and mCherry-Syk in RAW-Dectin1 macrophages at the indicated time points during co-incubation with β-glucan-coated beads (A), zymosan-Alexa647 (B), UV-killed *Candida*-BFP yeast (C) and UV-killed *Candida*-BFP hyphae (D). Experiments were performed multiple times, representive micrographs are shown.
(TIF)

Figure S2 **Localization of PI(4,5)P_2_ and PI(3,4,5)P_3_ during** ***C. albicans*** **phagocytosis.** PH-PKCδ-RFP and PH-BTK-GFP biosensors showing localization of PI(4,5)P and PI(3,4,5)P, respectively, without challenge or after 30 or 90 minutes of coincubation with *Candida*-BFP. White arrows indicate areas of PI(4,5)P_2_ and PI(3,4,5)P_3_ co-localization.
(TIF)

Figure S3 **Synthesis of PCI-32765.**
(TIF)
