# Supplementary figures and images for: Bruton's Tyrosine Kinase (BTK) and Vav1 Contribute to Dectin1-Dependent Phagocytosis of Candida albicans in Macrophages
Source: PLoS Pathog. 2013 Jun 27;9(6):e1003446. doi: 10.1371/journal.ppat.1003446 (PMC3694848; doi:10.1371/journal.ppat.1003446)

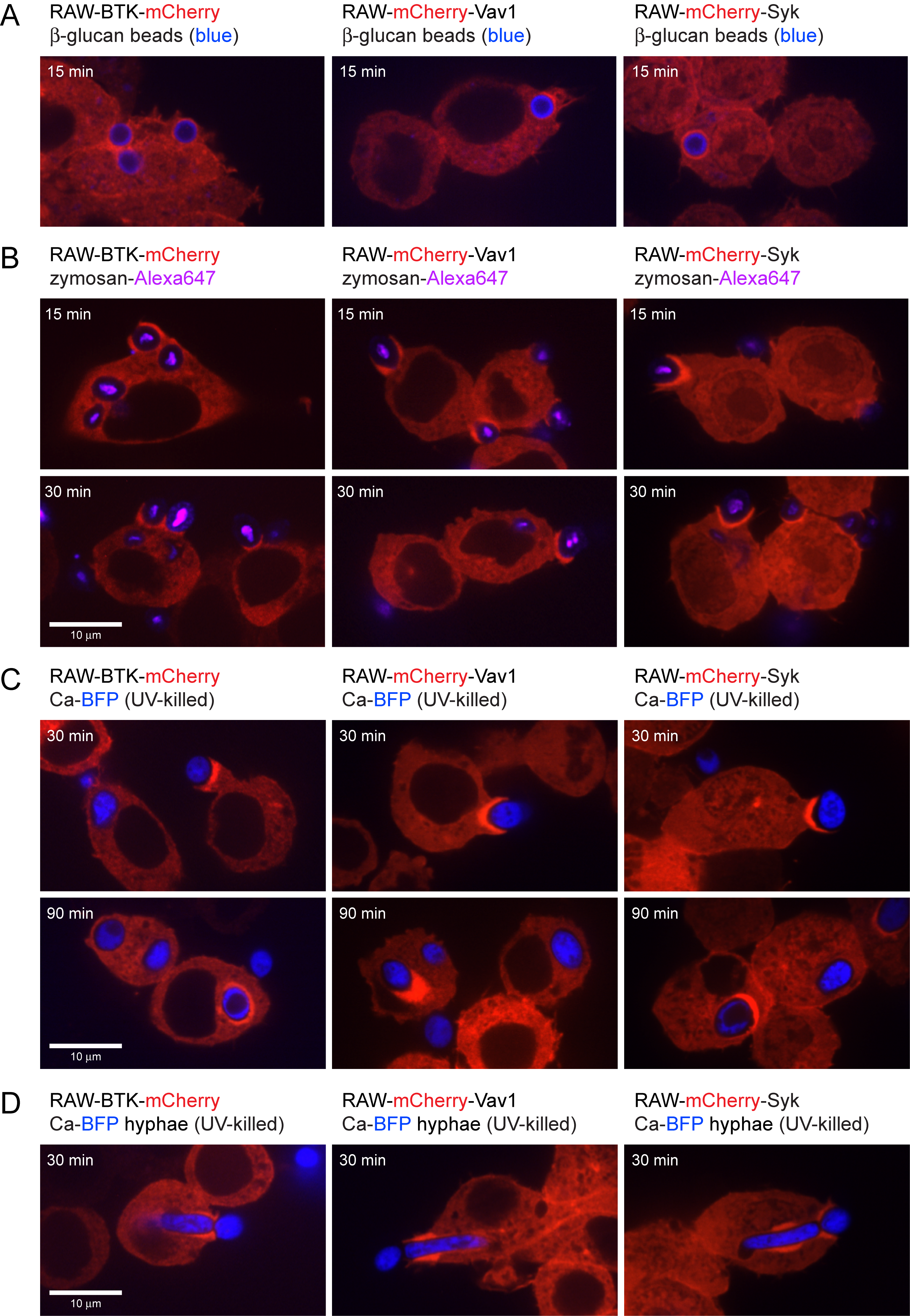

Supplement: Figure S1 — Localization of BTK-mCherry and Vav1-mCherry to the phagocytic cup. Confocal images showing localization of BTK-mCherry, mCherry-Vav1 and mCherry-Syk in RAW-Dectin1 macrophages at the indicated time points during co-incubation with β-glucan-coated beads (A), zymosan-Alexa647 (B), UV-killed Candida-BFP yeast (C) and UV-killed Candida-BFP hyphae (D). Experiments were performed multiple times, representive micrographs are shown. (TIF) [file ppat.1003446.s001.tif]

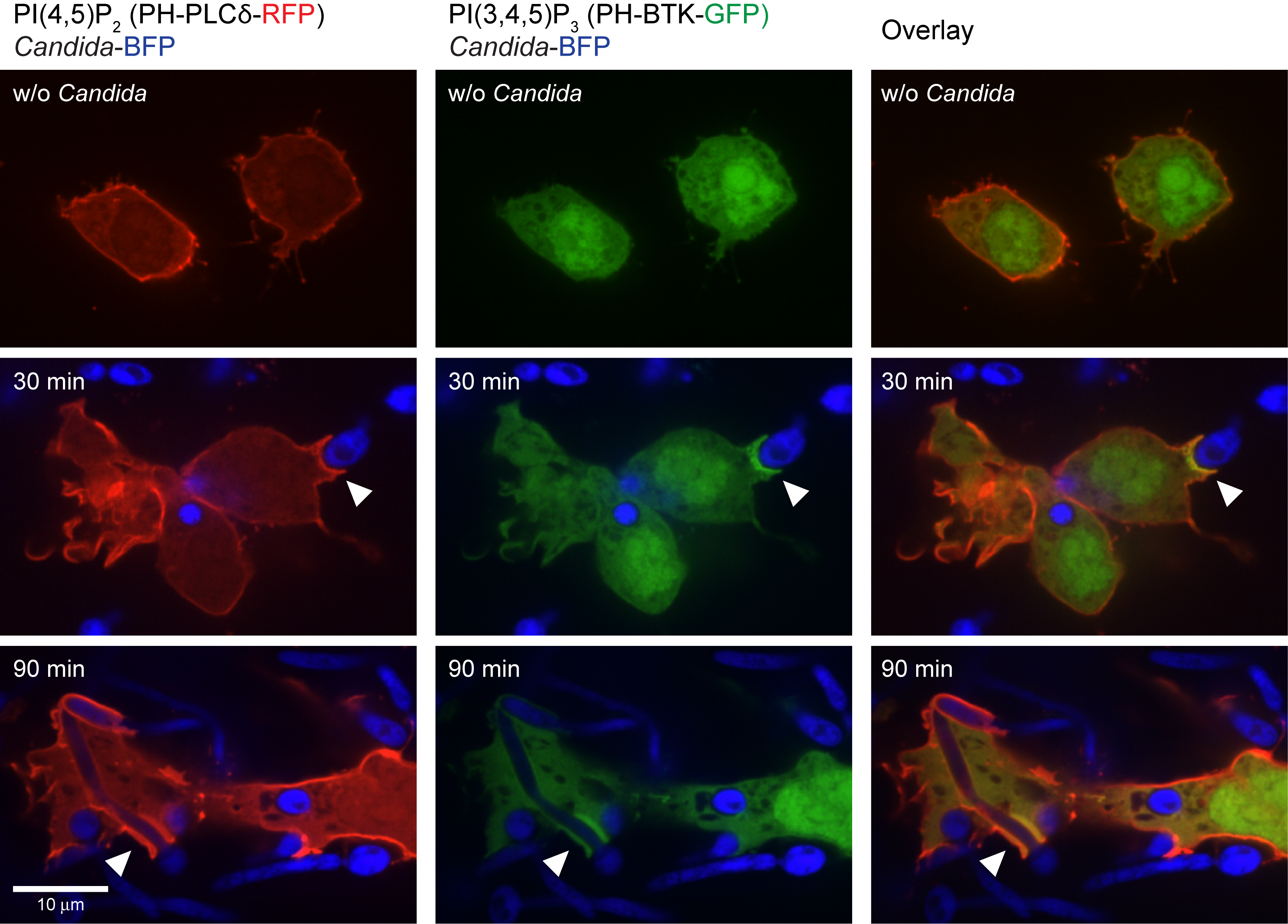

Supplement: Figure S2 — Localization of PI(4,5)P2 and PI(3,4,5)P3 during C. albicans phagocytosis. PH-PKCδ-RFP and PH-BTK-GFP biosensors showing localization of PI(4,5)P and PI(3,4,5)P, respectively, without challenge or after 30 or 90 minutes of coincubation with Candida-BFP. White arrows indicate areas of PI(4,5)P2 and PI(3,4,5)P3 co-localization. (TIF) [file ppat.1003446.s002.tif]

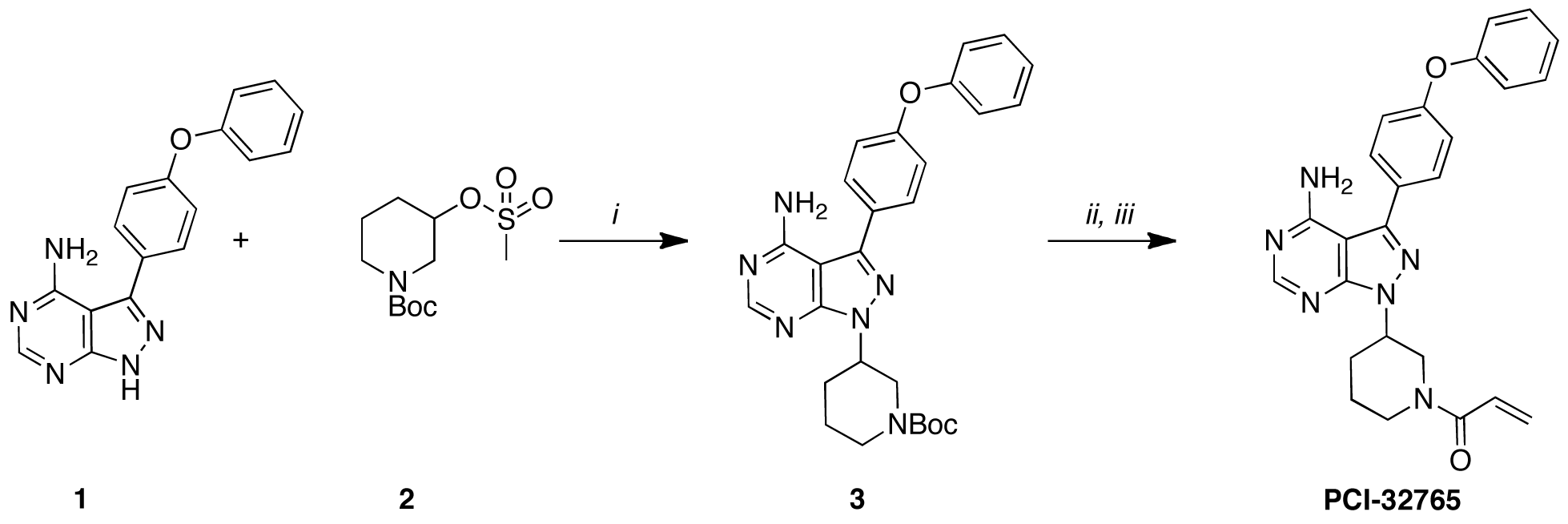

Supplement: Figure S3 — Synthesis of PCI-32765. (TIF) [file ppat.1003446.s003.tif]
